# Exploring Inflation-Related Public Discourse Relevant to Social Determinants of Health Using Social Media Data

**DOI:** 10.3390/ijerph23060694

**Published:** 2026-05-24

**Authors:** Yifan Zhang, Nethra Sambamoorthi, R. Constance Wiener, Hao Wang, Chan Shen, Sophie Mitra, Patricia A. Findley, Usha Sambamoorthi

**Affiliations:** 1 Department of Pharmacotherapy, College of Pharmacy, University of North Texas Health Science Center, Fort Worth, TX 76107, USA; nethra.sambamoorthi@unthsc.edu (N.S.); usha.sambamoorthi@temple.edu (U.S.); 2 Dental Public Health and Professional Practice, West Virginia University, Morgantown, WV 26506, USA; rwiener2@hsc.wvu.edu; 3 Emergency Medicine, JPS Health Network, Fort Worth, TX 76104, USA; hwang@ies.healthcare; 4 Department of Surgery, Pennsylvania State University, Hershey, PA 17033, USA; cshen@pennstatehealth.psu.edu; 5 Department of Economics, Fordham University, Bronx, NY 10458, USA; mitra@fordham.edu; 6 School of Social Work, Loyola University Chicago, Chicago, IL 60611, USA; pfindley@luc.edu; 7 Department of Pharmacy Practice, School of Pharmacy, Temple University, Philadelphia, PA 19140, USA

**Keywords:** social determinants of health, inflation, public health stressor, social media, sentiment analysis, content analysis, AI validation

## Abstract

**Highlights:**

**Public health relevance—How does this work relate to a public health issue?**

- It explores inflation as an economic stability social determinant of health by examining public narratives of inflation on social media platforms.
- It characterizes the digital social environment that can shape population health behaviors and receptivity to health-relevant policy.

**Public health significance—Why is this work of significance to public health?**

- Online discussion was predominantly negative and framed inflation at a macro-level rather than lived experience, an important signal for understanding community-level stress regarding inflation.
- It explores the methodological value of a human–AI approach to the digital public health surveillance of inflation.

**Public health implications—What are the key implications or messages for practitioners, policy makers and/or researchers in public health?**

- Practitioners and policy makers should anticipate that the inflation narrative is highly politicized and distrust-laden; effective messaging must explicitly address institutional trust and community-wide anxiety linked to economic stressors.
- Researchers should integrate validated social media analytics with surveys/interviews to capture underrepresented personal experiences of inflation-related strain and more accurately monitor the psychological burden of inflation as an SDOH.

**Abstract:**

Inflation, recognized as a social determinant of health (SDOH), significantly affects the daily lives of individuals through the rising costs of food, housing, and other basic needs, all of which are public health concerns. Since the COVID-19 pandemic, inflation has become a prominent concern in the U.S. and has been linked to increased stress and poor mental health among adults. While data on inflation is tracked routinely, how it is discussed publicly is understudied. Social media platforms provide insights into how inflation is framed and experienced by the public, and these assessments may be used to determine public health needs and policy advocacy. In this study, we conducted a time-bound, platform-specific case study of inflation-related discourse on X (formerly Twitter). Analysis revealed a predominance of negative sentiments (68.5%) including frustration and distrust. Posts primarily concerned monetary policy/government spending (31.6%), Federal Reserve interest rates/financial markets (24.5%), and U.S. presidential politics (12.9%). The users did not explicitly discuss personal-level hardships, and the discussions largely focused on macro-level issues framed in polarized political perspectives. These patterns matter for public health because institutional trust shapes support for social and health policies. Our study findings suggest a fragmented social environment that may exacerbate community-wide anxiety and challenge health promotion efforts and the need for public health surveillance through surveys or personal interviews to identify and address the psychological burden of inflation.

## 1. Introduction

Since 2021, inflation has emerged as a defining socioeconomic challenge with profound implications for population well-being. With the Healthy People 2030 framework, economic stability is recognized as a primary social determinant of health (SDOH), directly impacting the social and community context in which individuals live and function. As a dimension of economic stability, inflation directly erodes purchasing power, affecting individuals’ ability to secure basic needs such as food, housing, transportation, childcare, and medications, which are closely linked to well-established public health outcomes, including chronic disease management, mental health burden, and healthcare access disparities. In this way, inflation functions as a social determinant whose downstream behavioral and clinical consequences have measurable implications for population health outcomes. Recent empirical studies utilizing the United States (U.S.) Household Pulse Survey data revealed that over three-quarters of adults reported significant stress due to rising prices, a factor increasingly associated with clinical anxiety and depression [1,2]. While these studies establish the relevance of inflation within the SDOH framework, they primarily rely on structured survey data and macroeconomic indicators. Such approaches, although valuable, provide limited insight into how individuals experience, interpret, and communicate inflation in their daily lives. This gap is important because, from a theoretical perspective, the health relevance of economic conditions can be determined by objective exposure, subjective perception, and social interpretation.

To address this gap, this study draws on Stress Process Theory, which conceptualizes economic hardship as a chronic stressor that influences psychological well-being through perceived strain and uncertainty [3]. In parallel, the Social Ecological Model highlights that health-related behaviors and perceptions are shaped by individual factors and broader social and community contexts [4]. Within this framework, public discourse, particularly on digital platforms, can be understood as a collective environment where economic stress is interpreted, negotiated, and amplified.

Despite improvements in macroeconomic indicators since 2023 [5], public perceptions remain sharply discordant with data. This dissonance suggests that behavioral factors, such as financial literacy gaps and cognitive biases, contribute to a persistent state of economic anxiety among the population. From a public health perspective, how people perceive and interpret economic conditions is consequential as they can influence trust in institutions and support for policy interventions. When policy discourse prioritizes public sentiment over factual indicators, understanding the popular narratives that drive this community-wide stress is essential for effective health promotion. These narratives act as environmental stressors that influence health-seeking behaviors, such as the documented trend of individuals delaying medical care due to inflation-related financial concerns [1,2,6]. Thus, inflation-related discussions form part of the broader social environment that shapes health behaviors and policy contexts. By investigating these narratives, we can better understand the social and community context that either fosters resilience or exacerbates psychological distress.

Most research on people’s concerns related to inflation used surveys. For example, Pew Research collected information on whether people think inflation is a “very big problem,” with 62% citing inflation as a very big problem in 2024 [7], and Gallup tracked inflation as a top public concern [8]. But these data miss how ordinary people talk about inflation online in real time. While these survey-based findings have been instrumental in quantifying the prevalence and correlates of stress due to inflation, they are collected at discrete time points using few standardized questions. Consequently, little is known about how individuals experience, express, and negotiate stress related to inflation in their daily lives. Particularly, personal narratives around inflation have yet to be identified and examined using alternative data sources. This gap limits our understanding of how inflation, as a social determinant of health, is publicly interpreted and debated in everyday discourse.

Methodologically, advances in artificial intelligence (AI) and natural language processing (NLP) have enabled the systematic analysis of large-scale unstructured text data. Prior studies have demonstrated the effectiveness of extracting economic signals from textual sources such as policy communications [9,10], as well as the growing utility of deep learning and NLP techniques in economic forecasting and analysis [11,12]. Building on this body of work, the present study applies a hybrid human–AI approach to analyze inflation-related discourse on social media.

Social media platforms like platform X offer a novel lens for analyzing these narratives, capturing the emotional contagion of negative affect (e.g., anger and fear) that traditional surveys may miss. While individual-level SDOH stratification (e.g., by income or age) is often precluded by platform metadata limitations, these digital spaces provide a vital proxy for the collective social environment. The contributions of this study are threefold. First, it provides a novel empirical perspective by examining inflation-related discourse as a component of the social environment relevant to SDOH without directly measuring health impact. Second, it integrates theoretical frameworks from public health and social psychology to conceptualize the pathway linking economic conditions, perceived stress, and collective narratives. Third, it explores the applicability of large language models (such as ChatGPT) for capturing context-dependent social discourse, contributing to the growing literature on AI-assisted public health surveillance.

To achieve these aims, we analyze inflation-related posts to (1) characterize prevailing sentiments and thematic patterns within public discourse and (2) explore the reliability of AI-based methods in capturing socially embedded narratives. By situating digital discourse within the broader SDOH framework, this study contributes to emerging efforts to incorporate real-time, unstructured data into public health research and monitoring.

## 2. Methodology

### 2.1. Data Collection and Description

Publicly available datasets related to conversations about inflation did not exist. Widely used resources (e.g., Financial PhraseBank, SemEval Twitter sentiment corpora, DocNow Tweet Catalog) were domain-mismatched and temporally misaligned with the pre-election period; thus, they were unsuitable for our research question. There was a need to create a domain- and time-specific corpus for this study.

Data were retrieved from publicly accessible posts on X (formerly Twitter) (X Corp. Bastrop, TX, USA) using the X Application Programming Interface (API) v2. To capture pre-election discourse on inflation, a one-week sample of English-language posts published between 29 April and 5 May 2024 was collected. The sample of posts under analysis is representative of posts during the week of 29 April to 5 May 2024. We treat this corpus as a bounded, platform-specific case study of discourse during this seven-day window. Media headlines around inflation could be major drivers of social media posts on inflation, especially around the release of new inflation estimates and related commentaries by policy makers and elected officials (e.g., the President, Treasury Secretary, or congressional leaders). To avoid picking up noise around such media headlines, we selected a week when there was no release of inflation-specific reports by the Bureau of Economic Analysis or the Bureau of Labor Statistics nor any inflation-focused speeches from major elected officials. While there was a Federal Reserve (Fed) Board meeting during that week, when the Fed noted that inflation was moving towards, but had not yet reached, the 2% target, such meetings tend not to be followed by the public but rather by experts in relevant fields in finance and economics. We noted in the limitations that the sample of posts may not be representative of posts at other points in time. Posts were obtained via Python 3.9 (Python Software Foundation, Wilmington, DE, USA) scripting with the tweepy and twarc packages, which facilitate compliance with X’s rate limits and data policies.

Because “inflation” can be used to refer to different things (e.g., economic inflation vs. metaphorical uses like “grade inflation”), a dual-phase query design was implemented to isolate discussions of macroeconomic inflation. In the first phase, we compared a focused query using the standalone term “inflation” with a broader hardship-oriented query containing terms related to rising prices and affordability, including phrases such as “price/cost increases,” “high price/cost”, “gas price” and “can’t afford”. The purpose of this exploratory step was to assess the trade-off between recall and precision. Although the broader query captured some posts describing household-level economic strain, it also produced a large volume of irrelevant, promotional, commercial, and contextually ambiguous posts, especially for broad terms such as “gas prices” and “can’t afford.” The focused “inflation” query produced a higher proportion of posts directly related to inflation discourse and was therefore retained for the final data collection. This decision prioritizes the precision and interpretability of the corpus while recognizing that it may exclude some hardship-related posts in which users discuss rising living costs without explicitly using the term “inflation.”

Due to the API access and rate limits, a stratified random sampling approach was applied: posts were retrieved during 11 randomly selected one-hour intervals distributed evenly across the 7-day study period. This mitigated temporal bias (e.g., peak activity hours) while ensuring computational feasibility. Reposts without original commentary were excluded to prioritize user-generated content.

Each post included metadata (account creation date, language, user-generated location) and content (text, hashtags, timestamps, mentions). Unique user identifiers and screen names were retained solely to deduplicate entries and validate API accuracy. All procedures adhered to X’s Terms of Service, Developer Agreement, and ethical guidelines for social media research, ensuring anonymity by aggregating demographic data and removing personally identifiable information.

The initial query retrieved 9895 posts, with 9695 retained for analysis after excluding posts without references to inflation. These posts originated from 8226 unique user accounts, revealing a highly decentralized discourse structure: 90.2% of accounts contributed only once (7419 posts), while a small fraction exhibited disproportionate activity. Specifically, 1.0% of accounts generated 6.9% of posts, with the most prolific user contributing 0.4% of the corpus (37 posts). Original posts constituted 20.4% (*n* = 1975) of the dataset, reflecting direct user-generated commentary. The majority (72.9%) were replies within threaded conversations. Quoted posts with added commentary accounted for 6.7%, indicating a selective amplification of existing content.

### 2.2. Data Preprocessing

The dataset was preprocessed to optimize analytical validity. First, posts were verified to include explicit references to inflation by retaining only posts containing the keyword “inflation” or its hashtags. Elements such as URLs, mentions, numbers, and non-alphabetic characters were removed to minimize noise in topic modeling. Hashtags were preserved for semantic context but stripped of the “#” symbol. The spaCy [13] and scikit-learn Python libraries [14] were used for text preprocessing, including lowercasing and tokenization, due to their widespread adoption in natural language processing (NLP) research and spaCy’s demonstrated high accuracy in token tagging [15]. Stopwords were removed using the English stopwords corpus prior to topic modeling [16] and to generate word cloud visualizations.

Computational workflows were distributed across environments to balance efficiency and scalability. Data retrieval and cleaning were executed in Python 3.9.0 (Windows OS) using the pandas and re packages. Topic modeling was conducted in Google Colab with Python 3.11.11 (Google LLC, Mountain View, CA, USA). Visualization was implemented in R 4.0.4 (R Foundation for Statistical Computing, Vienna, Austria) with the ggplot2 and wordcloud packages for graphical output. Code reproducibility was ensured through version-controlled scripts and environment snapshots.

### 2.3. Data Analyses

#### 2.3.1. Sentiment Analysis (All Posts)

A multi-method sentiment analysis was deployed to assess the results. First, we used SentiStrength, a validated lexicon-based classification tool for social media sentiment analysis [17], to assign dual polarity scores to each post. Positive sentiment was scaled from 1 (neutral) to 5 (extremely positive). Negative sentiment was scaled from −1 (neutral) to −5 (extremely negative). We then added these two values together to create a single composite score ranging from −4 (very negative) to +4 (very positive) and grouped posts into three categories: positive if the positive side was stronger; negative if the negative side was stronger; and neutral if they balanced out. The tool’s robustness stems from its hybrid lexicon–rule architecture, which accounts for informal grammar, slang, and emoticons [18] and is still adopted to address modern challenges like information extraction from big data [19]. However, because this study focused on inflation-related discourse, this lexicon-based approach may be sensitive to the repeated presence of negatively valenced economic terms such as “inflation.” For this reason, the SentiStrength results were interpreted as a corpus-level comparator rather than as a definitive classification of contextual sentiment in individual posts. Top frequency terms, as well as top frequency verbs and nouns, were examined from positive, negative, and neutral posts.

#### 2.3.2. Sentiment Analysis: Generative AI Tools (Subsample Posts)

We used ChatGPT, a large language model (LLM) AI tool, for sentiment classification [20]. Based on sample size calculation, as well as concerns of computational constraints, we selected a simple random sample of 200 posts from the full corpus without replacement for ChatGPT’s sentiment classification. We experimented with several ways of organizing prompts to conduct analyses. Initially, the prompt instructed ChatGPT to perform both tasks at once: “First, perform sentiment analyses on the following tweets related to inflation, categorize them into positive, negative, neutral, other (please specify other sentiments), can’t determine, or not relevant. Second, perform semantic analyses on these tweets.” However, this multi-task approach was later refined to improve clarity and performance by focusing on one task at a time. The final prompt for sentiment analysis was as follows:

“For each tweet, identify the overall sentiment expressed towards inflation and categorize it into one of the following categories:

- Positive: The tweet expresses a positive or favorable sentiment towards inflation.
- Negative: The tweet expresses a negative or unfavorable sentiment towards inflation.
- Neutral: The tweet does not express any clear sentiment towards inflation, and it is factual or objective in nature.
- Other (specify the sentiment): The tweet expresses a sentiment other than positive, negative, or neutral towards inflation. Please specify the sentiment, such as sarcastic, humorous, or confused.
- Can’t determine: The sentiment expressed in the tweet towards inflation is too ambiguous or unclear to categorize.
- Not relevant: The tweet does not mention or relate to inflation.

Provide a brief explanation or justification for your sentiment categorization for each tweet.”

The performance of ChatGPT sentiment analysis was assessed by comparing its output with SentiStrength via inter-method agreement and pairwise agreement. Detailed performance metrics of ChatGPT and SentiStrength (e.g., accuracy, recall, precision and F1 score) were reported using the human annotation as references. Metrics were calculated by sentiment category and summarized using macro-averaged performance to account for differences in category prevalence.

#### 2.3.3. Sentiment Analysis: Exploratory Human Validation Exercise (Subsample Posts)

We also explored how ChatGPT sentiment analysis compared with sentiments generated by human experts. For a random sample of 200 tweets, we compared ChatGPT sentiment analysis with sentiments generated by human experts. The goal of this analysis was to explore AI’s capacity to detect nuanced sentiments, such as feigned anger and guarded optimism, and to interpret pragmatic cues like sarcasm. Seven independent human experts annotated the 200 posts to establish reference labels. Final sentiment labels were determined by majority vote, with unresolved ties adjudicated by an arbitrator (PF). Inter-rater reliability was also evaluated using Krippendorff’s α (ordinal) for the negative, neutral, and positive sentiment categories, excluding other labels [21].

#### 2.3.4. Topic Modeling (All Posts)

Key themes were extracted through topic modeling for all posts. Topic modeling using non-negative matrix factorization (NMF) was applied to the TF-IDF vectorized corpus. Hyperparameters were tuned to maximize topic coherence scores [22]. The optimal number of topics k was determined by iterating k from 2 to 30 and selecting the model with the highest coherence score, a method validated in prior work [23,24].

#### 2.3.5. Content Analysis: Generative AI Tools (Subsample Posts)

We used ChatGPT-4o and ChatGPT-5.5 to generate summaries for the subsample posts. Emojis and all features of the posts were retained in the entirety of the textual examination. We experimented with several prompt designs to achieve consistent output and provide clear instructions for this content analysis. The initial prompt was as follows: “Identify and exact key themes, topics of each tweet. Provide an overall summary of themes from all tweets. Ensure that your analysis is thorough, objective, and well-supported by evidence from the tweets themselves.” This was followed by an expanded prompt for deeper linguistic and contextual interpretation: “Analyze the language used in the tweet, such as the choice of words, phrases, or metaphors, and how they convey meaning or connotations related to inflation. Identify any implicit or explicit assumptions, beliefs, or opinions expressed in the tweet about inflation. Analyze the context or background information that might be relevant for understanding the tweet’s meaning or sentiment towards inflation. Provide a summary or interpretation of the overall meaning and implications of each tweet related to inflation.” However, in early testing, we observed two issues that reduced reliability: ChatGPT-4o sometimes produced computer code rather than returning direct analytical results, and it tended to generate fabricated posts when asked to process 50 posts at once. To mitigate these issues, the final prompt was refined for clarity and control: “Perform semantic analysis on each of the posts and give one key theme of each post under 15 words. Please analyze the text directly one by one without external tools and work in smaller batches for processing and output.”

#### 2.3.6. Content Analysis: Human Validation (Subsample Posts)

Topic labels from topic modeling and AI-generated summaries were reviewed by the seven experts. In cases of disagreement, the panel engaged in open discussion to reach consensus; when full agreement was not achievable, decisions were made by majority vote. The level of agreement was categorized as full concordance (theme fully matched), partial concordance (theme partially matched), or discordance (no thematic overlap). Posts with discordant or partially concordant labels under ChatGPT-4o were subsequently refined using ChatGPT-o1 preview to improve thematic interpretations.

#### 2.3.7. Identification of Health-Related Posts

Health-related posts were identified by a list of keywords and analyzed using qualitative content analysis. Keywords included health, medical, illness, stress, anxiety, depression, insomnia, psychological, insulin, opioid, fentanyl, and so on. Posts were reviewed to assess whether they expressed a substantive connection between inflation and health or only mentioned health-related terms incidentally or as part of a broader political statement. Posts were interpreted as expressions of public perception and lived concern. This approach allowed the analysis to identify how inflation was perceived as affecting physiological and psychological health.

### 2.4. Research Team and Qualitative Analysis

Inflation is not solely an economic phenomenon; it has wide-ranging implications for access, health, equity, and population well-being. To capture this complexity in our analysis, we deliberately assembled an interdisciplinary annotating team. Economists contributed to domain-specific knowledge about inflation and price dynamics. Other experts with training in medicine, dentistry, epidemiology, and public health brought perspectives on how inflation intersects with health systems, access to care, and population health outcomes. Biostatisticians and health services researchers helped us with measurements, the selection of posts for annotation, and visualization. The interdisciplinary group ensured that the annotations captured not only economic dimensions but also the social and health consequences of inflation, a social determinant of health. Although our study did not directly examine the health consequences of inflation, the inclusion of such expertise was integral to producing a more comprehensive and methodologically rigorous interpretation. This approach is consistent with established best practices in qualitative content analysis and mixed-methods social media research [25].

To further address potential interpretive bias—a recognized limitation in qualitative analysis [26,27]—each researcher documented their positional identity, including disciplinary training, career stage, geographic location, institutional affiliation, and cultural background. This practice fostered analytical transparency and reflexivity throughout the process. The annotation team included professionals working in the U.S., born in different countries (France, India, U.S., and China) with diverse cultural backgrounds, trained in economics, medicine, dentistry, epidemiology, biostatistics, public health, and health services/outcomes research. Geographically, members were drawn from urban, rural, and metropolitan settings. In terms of career stage and demographics, representation ranged from early-career scholars to senior and emeritus faculty. While shared academic perspectives and prior collaborations promoted team cohesion, independent data analysis was enforced to minimize groupthink.

Methodologically, sentiment annotation was conducted completely independently: each researcher first labeled data without consultation, and disagreements were later resolved through structured team debates to reach consensus on final sentiment classifications, enhancing validity and consistency [28]. Content analysis validations were determined collaboratively through open discussion among the research team to achieve shared interpretation. Sentiment and topic validations underwent triangulation between manual coding and algorithmic outputs, cross-verifying human and computational interpretations [29]. This mixed-methods approach balanced qualitative depth with computational scalability, aligning with best practices for social media research [30].

## 3. Results

**Figure 1:**
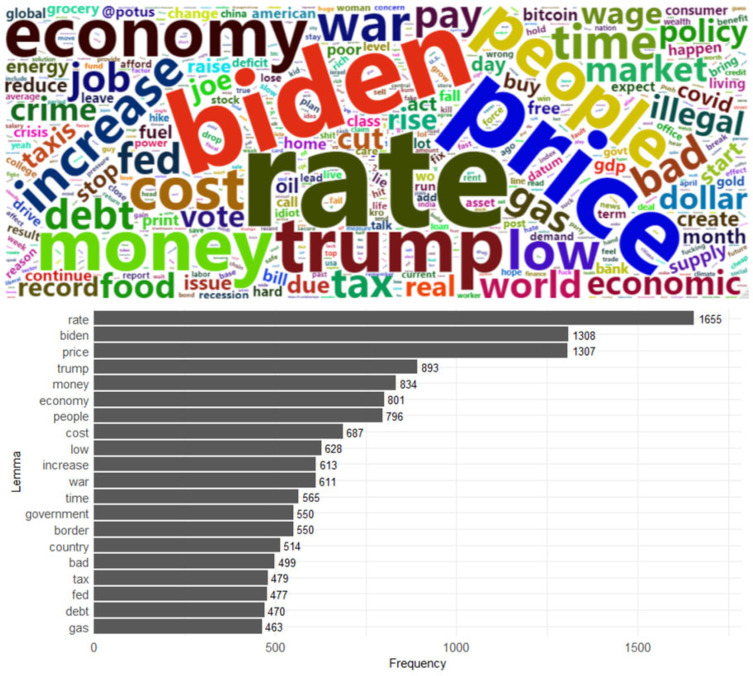
Top 20 most frequent lemmas and word cloud of inflation-related posts from social media platform X.

The corpus (*N* = 9695) contained significant political content, with explicit mentions of Biden (1308 mentions) and Trump (893 mentions) collectively comprising 20% of posts. Lexical frequency analysis identified salient economic terms (Figure 1), including “price” (*n* = 1307), “money” (*n* = 834), and “economy” (*n* = 801), which clustered around fiscal policy and consumer strain.

The 10 most frequent hashtags were inflation, bitcoin, fed, economy, nigeria, fomc, gold, btc, interestrates, and trump2024. Three fields were highlighted, including economic policy (#fed, #fomc, #interestrates, #economy), financial markets (#bitcoin, #btc, #gold) and political discussions (#trump2024).

### 3.1. Sentiment Analysis

**Figure 2:**
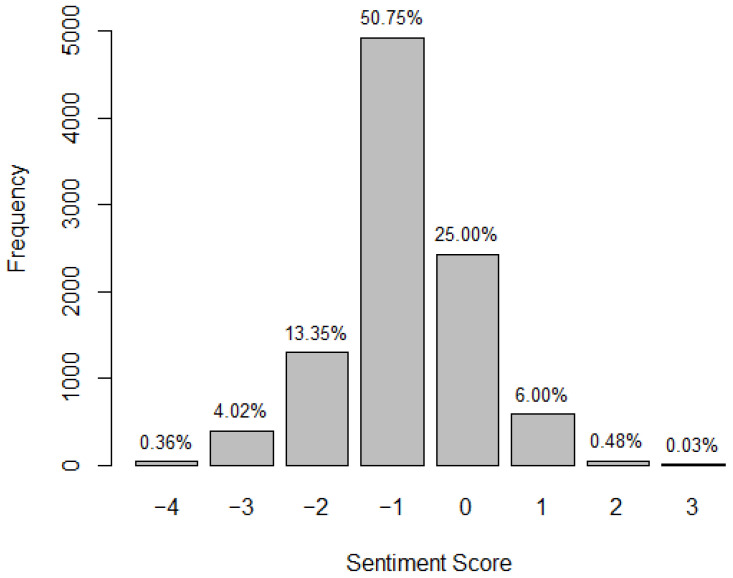
Distribution of sentiment scores from inflation-related posts from social media platform X.

The analyses revealed a predominance of negative sentiment (Figure 2), with 68.5% of posts classified as negative, followed by neutral (25.0%) and positive (6.5%) posts. To investigate thematic differences among sentiment categories, the most frequent lemmas within each group were extracted. Among negative posts, the top five lemmas were “biden,” “rate,” “price,” “trump,” and “war.” In contrast, positive/neutral posts featured similar high-frequency lemmas, including “rate,” “price,” “biden,” “economy,” and “money.” Notably, exclusively positive posts (6.5% of the corpus) also demonstrated overlapping terminology, with “rate,” “economy,” “price,” “america,” and “trump” as the most frequent lemmas. A further linguistic analysis of verb and noun usage across sentiment categories yielded comparable results, suggesting no significant divergence in topical focus between negative and positive/neutral posts.

### 3.2. Topic Modeling

**Figure 3:**
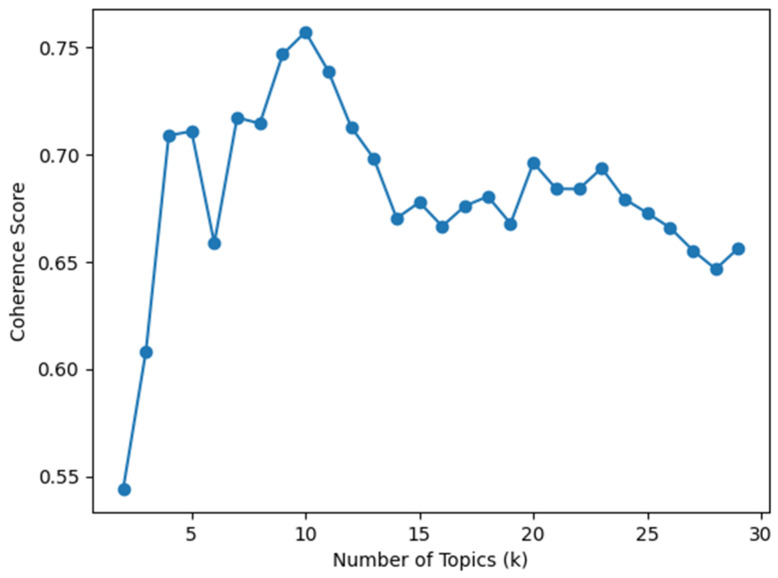
Coherence scores for topic models with varying numbers of topics (k) applied to inflation-related posts on platform X, May 2024.

Topic modeling via NMF was applied to identify latent themes within the corpus. Model selection was guided by optimizing the coherence score, with a configuration of k = 10 topics yielding the highest coherence (Figure 3). The final model identified ten distinct topics, ranked by their proportional representation in the corpus (Table 1). The top five topics were Money Policy and Government Spending (31.6%), Federal Reserve Interest Rates and Financial Markets (24.5%), U.S. Presidential Politics and Pandemic-Era Economy (12.9%), Inflation and Border Security in Political Rhetoric (9.4%), and Global Energy Markets and Geopolitical Conflict (5.7%).

### 3.3. Human Annotation and Model Agreement

Inter-annotator concordance on initial sentiment classification was observed in 24% of the posts (*n* = 47), while 77% (*n* = 153) required majority voting or arbitration. Krippendorff’s α for negative, neutral, and positive sentiments (excluding other labels) was 0.712 (95% CI = [0.625, 0.790]), indicating a moderate level of consistency. As shown in Table 2, the final human-annotated sentiment distribution was: 5% positive, 39% negative, 19% neutral, and 21% not relevant (e.g., posts such as “Social Security COLA is fraudulent government inflation numbers” were deemed unrelated to inflation sentiment). Furthermore, the same 200 posts were analyzed for sentiment classification by ChatGPT and SentiStrength. ChatGPT’s classifications aligned with the human annotators’ final labels in 57% of cases, whereas SentiStrength agreed with the human labels in only 33% of cases. The validation results indicate that the lexicon-based approach tended to identify a larger share of posts as negative than was observed in the human-annotated validation subset. This discrepancy suggests that SentiStrength may be sensitive to the negative valence of inflation-related terminology itself, whereas human coders and the AI-based approach were better able to account for the broader context of each post. Moreover, SentiStrength was unable to categorize sentiments in a way other than positive, neutral or negative. Accordingly, the SentiStrength findings should be interpreted as a supplementary corpus-level indicator rather than as the primary measure of sentiment. Similarly, performance metrics using the human annotations as the standard showed that ChatGPT performed substantially better than SentiStrength overall, especially on macro F1: 0.518 vs. 0.211. ChatGPT also has higher accuracy: 57.0% vs. 33.0%. After removing posts labeled as other, ChatGPT still performed better than SentiStrength (Table 3).

Thematic concordance between human experts and ChatGPT revealed an 82% agreement (full concordance) in thematic classification. ChatGPT demonstrated strong performance in thematic tasks, significantly outperforming topic modeling (40% agreement). It was also capable of identifying a sarcastic tone. For instance, a post like “Too bad we had a pandemic, and inflation was predicted worldwide because of supply chain issues. Luckily, we’re too smart to blame it on a human being since it was a pandemic. Thank God we’re intelligent.” was recognized as sarcastic by ChatGPT. For the 12 posts exhibiting discordance or partial concordance by ChatGPT-4o, refinement using ChatGPT-o1 preview was applied, resulting in 100% full concordance with human validation. For instance, a post that read “If you think Joe Biden is at fault for our inflation, you’re an idiot”—initially summarized as “Criticism of Joe Biden regarding inflation” by ChatGPT-4o—was correctly identified as “strongly rejecting the notion that Biden is responsible for inflation”. Similarly, a post stating “pay wet inflation aim town unlike” was correctly labeled as “nonsensical content” after applying ChatGPT-o1 preview. However, despite ChatGPT’s high performance, computational limitations precluded its application to the full corpus.

### 3.4. Health-Related Posts

There were 361 (3.7%) posts identified by health-related keywords, among which 79 were highly relevant with a strong implication of health risk due to inflation; 114 were moderately relevant, linking inflation to health through policy, systems, or co-occurring crises; 157 were of low relevance, with political arguments and rhetorical health language; and 11 were irrelevant after manual review. Similarly to the full corpus, the health-related subcorpus contained many political or rhetorical posts. In particular, 43.5% mentioned inflation alongside topics such as crime, immigration, abortion, drug use, or mental health-related insults. Nevertheless, among the moderately and highly relevant posts, several substantive themes emerged, including healthcare affordability and medication and insurance costs; income erosion, poverty, and social protection; food and nutrition insecurity; psychological distress, stress, depression, and burnout; healthcare system strain; and housing insecurity and utility costs. In addition, 85 posts mentioned health and inflation together without specifying a mechanism or explicit pathway linking inflation to health outcomes. Other posts expressed concern, complaints, or anger about care costs, income erosion, the substance use crisis, and inadequate social protection.

## 4. Discussion

It is well established that economic factors have a major impact on health, in particular unemployment and poverty. At a macro-level, there is limited evidence on whether and how inflation may impact health as inflation has in general not been a significant risk since the 1980s. Since the COVID-19 pandemic, inflation has become a major economic concern, and in light of recent studies pointing at inflation as a significant stressor for the majority of Americans [1,2], we set out to conduct a time-bound, platform-specific case study of inflation-related discourse on X and explore the feasibility of using AI-assisted text analysis to characterize social media narratives related to economic stressors.

We analyzed 9695 inflation-related posts on platform X to decode inflation narratives while evaluating AI’s role in social media discourse analysis. The high proportion of conversational engagement (72.9% replies, 6.7% quoted posts) underscores inflation’s role as a catalyst for public discourse, aligning with prior work linking crises to participatory communication [31]. Analysis revealed three dominant discourses: (1) economic policy debates (#fed, #interestrates), reflecting the scrutiny of institutional trust; (2) financial market narratives (#bitcoin, #gold), signaling skepticism toward traditional fiscal safeguards, a phenomenon tied to post-2008 inflationary anxiety [32]; and (3) partisan political rhetoric (#trump2024), weaponizing inflation as an electoral wedge issue, mirroring politicized health [33] and climate debates [34]. The prominence of #nigeria indicated transnational inflation anxieties in emerging economies, a theme underexplored in U.S.-centric frameworks but critical in globalized systems. These findings illustrate how inflation discourse intersects with institutional trust, market behavior, and polarization while revealing global linkages. Because economic instability (in our context: inflation) is a core SDOH, these patterns are relevant beyond political communication: they reflect how a structural driver of health is publicly interpreted and contested.

Among the health-related posts, inflation was represented as a set of material constraints affecting health and well-being. Users described medical bills, neonatal intensive care stays, insurance premiums, medication prices, and hospital budgets as points at which illness could become financially catastrophic. They also linked groceries, “healthy foods,” salmon, bread, food bank lines, and fast food prices to diet quality and food insecurity. Housing-related costs, including rent, mortgages, utilities, and homelessness, were connected to the erosion of safe shelter and family stability. These discussions suggested several potential pathways from inflation to health risk, including delayed or foregone treatment, medication nonadherence, unmanaged chronic disease, poorer nutrition, exposure to inadequate housing or utilities, and reduced service capacity when hospitals or health workers could not keep pace with rising costs and wages. Psychologically, users repeatedly associated price pressure with stress, anxiety, depression, burnout, despair, and domestic conflict. These posts therefore showed how inflation could be experienced not only as a temporary budget convenience but also as a chronic source of health-related insecurity. Although they represented only a small proportion of the corpus, these posts collectively revealed a plausible social determinant mechanism through which rising prices compressed household choices, undermined access to care and nutrition, intensified housing insecurity, and translated economic precarity into concrete health risks.

Additionally, sentiment analysis revealed substantial lexical overlap between positive and negative posts (e.g., shared terms like “biden,” “price”), suggesting that expressed sentiment was driven more by polarized frames than by topic-specific critique. This pattern aligns with Taylor’s [35] concept of performative rhetoric on social media, where users project ideological positions but seldom share authentic personal experiences. Critically, our data showed that this performative dynamic extended to inflation discourse: posts framed inflation as a detached macroeconomic or political symbol (here are two examples of such posts: (i) “Inflation is global, not just here in America. It’s actually greedflation. This was caused by Trump removing regulations that prohibited price gouging” and (ii) “Biden Climate Agenda is creating more inflation; more pain for Americans.”) while largely omitting stories of personal financial stress. From a public health perspective, this absence of lived-experience narratives is notable. If inflation functions as an SDOH, the limited visibility of personal coping or hardship may obscure how economic strain is experienced at the population level. Our findings indicate that platform X may function more as a site of political positioning than as a venue to articulate stress around inflation and coping strategies and receive or provide mutual support during periods of inflation.

Methodologically, this study advances social media textual analysis by exploring the benchmarking of AI tools. While lexicon-based tools like SentiStrength efficiently classify sentiment at scale, their reliance on predefined polarity (e.g., “inflation” coded as inherently negative) risks conflating factual discourse with affective stance. Because the corpus was constructed around inflation-related posts, many posts contained terms that are intrinsically negative or associated with economic strain. SentiStrength may therefore classify posts as negative based on the presence of words with negative emotional valence even when the post’s broader meaning is neutral, informational, sarcastic, or politically contextual. In contrast, ChatGPT achieved higher agreement with human annotations (57% sentiment, 82% thematic alignment) by interpreting context-dependent cues like sarcasm (e.g., “I didn’t know the nazis had low inflation, low gas prices, high employment, strong economy, no wars and a powerful military?”). This underscores AI’s semantic flexibility in accommodating political–economic pragmatics [36], positioning them as promising tools for scaling qualitative analysis. For public health research, this has practical implications: accurately capturing tone and context is essential when monitoring digital discourse around SDOH such as inflation, where misclassification could distort assessments of public concern or polarization.

Our human–AI approach, using tailored ChatGPT prompting, revealed nuanced sentiments, such as feigned anger and guarded optimism. This demonstrates AI’s growing capacity to interpret pragmatic cues like sarcasm without explicit guidance [37]. However, scalability constraints necessitated Python-based tools for large corpora, highlighting inherent trade-offs between AI’s precision and efficiency. Critically, these findings challenge assumptions that AI can supplant human judgment in decoding affective complexity. We suggest further exploring a framework where AI handles pattern recognition and humans validate contextual nuance. Future improvements may involve fine-tuning AI on domain-specific corpora to increase accuracy and reduce hallucinations [38,39] and expanding multi-platform data sources (e.g., YouTube, Reddit) to enhance demographic representation. Hybrid pipelines combining AI’s contextual reliability with traditional NLP’s scalability warrant further exploration. Such approaches may strengthen emerging efforts to integrate digital discourse analysis into the public health surveillance of economic and policy-related stressors.

Overall, our approach explored a hybrid human–AI validation approach to examining stress due to inflation on social media. First, we collected a focused set of posts about inflation during the U.S. pre-election period, sampling across different time points so that the data reflected the broader conversation rather than a single moment. Second, we compared how traditional lexicon-based tools and a generative AI model classified sentiment, using human coders as the benchmark. This provides a transparent test of where AI adds value and where it falls short. Third, we introduced a simple way to check whether political polarization stems from people talking about the same topics with different emotions or from people talking about entirely different topics. This “different sentiments, shared topics” perspective links computational results to theories of narrative economics and moves beyond the limitations of word clouds. Taken together, these steps improve the validity and interpretability of social media analysis, and they provide a model for studying other high-stakes public policy debates. Because macroeconomic conditions such as inflation operate as SDOH, improving analytic tools for interpreting related discourse has direct relevance for population health research.

This study has several limitations. First, platform selection bias is likely. Users of X skew toward politically engaged populations and exclude those without digital access and those disinclined toward online discourse—a selection bias endemic to computational social science using social media data. Different demographic profiles [40] and declining uptake among U.S. adults [41] further limited demographic generalizability. Second, limited metadata constrained subgroup analyses. The absence of granular user attributes (e.g., age, income) precluded a systematic assessment of how inflation narratives may vary across socioeconomic strata. This is particularly relevant given that the health impacts of inflation are unevenly distributed across income and demographic groups [1,2]. Third, the authenticity of accounts is uncertain. Since November 2022, the blue “verification” badge primarily indicates a paid subscription rather than identity verification; thus, accounts may be organizational, automated, or pseudonymous and not necessarily individual humans. Relatedly, social bots and automated accounts are a known presence on X [42] and can influence diffusion dynamics and content visibility [43,44,45,46]. Their activity can therefore influence corpus composition and the sentiment landscape observed here. Fourth, this analysis is cross-sectional within a defined study window of a particular week, so the findings should not be assumed to be generalizable across other inflation news cycles or policy moments. In addition, we were unable to perform diachronic analysis to track how narratives “mutate” over time and across events or policy cycles. Our comparison of ChatGPT sentiment analysis with sentiments generated by human experts was limited to an exploratory analysis of 200 tweets. Fifth, the final dataset was collected using the keyword “inflation.” Although exploratory testing showed that broader affordability-related keywords introduced substantial noise, the use of a focused keyword may bias the corpus toward users who explicitly frame economic hardship using macroeconomic language. As a result, the dataset may underrepresent posts from individuals who experience inflation primarily as everyday survival stress and describe it through terms such as rent, groceries, gas prices, bills, or inability to afford basic needs. Future research could combine keyword expansion with supervised classification or manual validation to better capture these individual-level hardship narratives while filtering promotional and irrelevant content. Finally, while ChatGPT outperformed SentiStrength, its “black-box” architecture obscures sentiment attribution logic, complicating reproducibility. Hallucinations (e.g., fabricating posts during batch processing) and opacity underscore the need for improved transparency and bias mitigation protocols, necessitating cautious application in policy-sensitive domains [47,48].

## 5. Conclusions

In summary, this study analyzed inflation narratives on social media. Our findings suggest that social media, in its current state, may exacerbate community-wide inflation-related anxiety through emotional contagion rather than fostering social support. As inflation is an SDOH, understanding how it is framed and politicized in digital spaces is important for public health research and policy planning. Given the underrepresentation of personal narratives due to inflation in digital spaces, future research must employ targeted surveys and in-depth interviews to identify suppressed personal narratives. The continuous integration of real-time digital surveillance with national data, such as the Household Pulse Survey, is essential to identify and mitigate the psychological burden of economic stressors and promote population-wide mental health resilience in increasingly digitized societies. Integrating digital discourse analysis with traditional epidemiologic data may strengthen efforts to monitor and respond to the health implications of macroeconomic factors such as inflation.

## Figures and Tables

**Table 1:** Non-negative matrix factorization (k = 10) topic model of platform X (formerly Twitter) discourse (*N* = 9695 posts), ranked by percentage.

Topic	Prevalence (%)	Thematic Label	Salient Features (Top 10 Terms)
0	31.6%	Money Policy and Government Spending	money, like, people, just, government, dont, tax, spending, printing, know
7	24.5%	Federal Reserve Interest Rates and Financial Markets	rates, rate, fed, higher, growth, cut, year, real, market, lower
3	12.9%	U.S. Presidential Politics and Pandemic-Era Economy	trump, biden, economy, president, border, wars, covid, amp, pandemic, world
2	9.4%	Inflation and Border Security in Political Rhetoric	high, prices, want, open, gas, borders, wars, groceries, vote, crimes,
9	5.7%	Global Energy Markets and Geopolitical Conflict	oil, gas, day, joe, world, barrel, wars, end, attacking, skyrocketed
6	4.3%	Nigerian Infrastructure and Economy	nigeria, cost, amp, reticulated, transporting, seaports, completed, speedy, execution, connect
8	4.0%	Anti-Immigration Sentiment and Political Criticism	bad, caused, illegal, bidens, sucks, aliens, jihadists, idiots, america, reign
1	3.1%	Legislative Spending Debates	act, reduction, democrats, billion, cost, saying, claim, breaking, living, infrastructure
4	2.4%	Illegal Immigration and Community Impact	illegals, year, bounty, beautify, civilian, neighborhoods, solution, stimulate, hunter, safe
5	2.1%	Media Discourse and Inflation	say, dstv, transitory, dont, higher, hes, idea, hi, works, okay

Topics ordered by descending prevalence percentage. Salient features show the highest weight terms from the NMF model (stopwords removed). Thematic labels are assigned through the qualitative coding of term sets and representative posts. Model coherence score: 0.76.

**Table 2:** Comparison of annotation methods: sentiment distribution, agreement, and theme concordance (N = 200).

Sentiment Annotations Across Methods			
Sentiment	SentiStrength	ChatGPT *	Human *
Positive	20 (10%)	12 (6%)	9 (5%)
Neutral	41 (21%)	45 (23%)	37 (19%)
Negative	139 (70%)	104 (52%)	77 (38.5%)
**Inter-Method Agreement of Sentiment Analysis Across Methods**			
Agreement Level	Number of Posts (%)		Examples
Full Agreement (All three methods)	54 (27%)		“*Why do you Democrats encourage more inflation? Biden Climate Agenda is creating more inflation, more pain for Americans.*”
Pairwise Agreement Only			
SentiStrength and ChatGPT	40 (20%)		*“I have been thinking a lot about this. If the money is broken it doesn’t matter who we vote for because QE and inflation centralizes power”* (negative vs. neutral by human annotation)
ChatGPT and Human	60 (30%)		*“There are historical reasons but ultimately, it’s a political decision to do it this way and there are no laws of economics, finance, or accounting that make lending necessary for currency. See the Buckaroo fiat currency going strong for years and zero inflation”* (neutral vs. negative by SentiStrength)
SentiStrength and Human	12 (6%)		NA
**Theme Concordance**			
Comparison	Discordance	Partial Concordance	Full Concordance
Human vs. ChatGPT	18 (9%)	18 (9%)	164 (82%)
Human vs. Topic Modeling	68 (34%)	52 (26%)	80 (40%)

Example posts are edited in terms of spelling and redacted information to protect re-identification. * Numbers may not add up due to other categories included, other, can’t determine, and not relevant, which are not shown.

**Table 3:** Performance metrics for sentiment classification using ChatGPT and SentiStrength, with 4-category versus 3-category evaluation.

Method	Accuracy	Macro Precision	Macro Recall	Macro F1	Weighted Precision	Weighted Recall	Weighted F1
4-Category Evaluation on Positive, Neutral, Negative, and Other Cases							
ChatGPT	0.570	0.519	0.556	0.518	0.574	0.570	0.546
SentiStrength	0.330	0.170	0.288	0.211	0.194	0.330	0.243
3-Category Evaluation on Positive, Neutral and Negative, Excluding Other Cases							
ChatGPT	0.740	0.782	0.642	0.702	0.846	0.740	0.789
SentiStrength	0.537	0.367	0.384	0.371	0.517	0.537	0.523

## Data Availability

The raw X data analyzed in this study are subject to the platform’s Terms of Service and Data Use Agreement, which restrict the redistribution of third-party content. To ensure compliance with these terms, the dataset cannot be publicly shared. Researchers may request access to comparable data directly through X’s official API or data licensing portal (https://developer.x.com). Aggregated or anonymized summaries of the data, as well as code for analysis, are available from the corresponding author upon request.
